# Linking household survey and health facility data for effective coverage measures: a comparison of ecological and individual linking methods using the Multiple Indicator Cluster Survey in Côte d’Ivoire

**DOI:** 10.7189/jogh.08.020803

**Published:** 2018-12

**Authors:** Melinda K Munos, Abdoulaye Maiga, Mai Do, Glebelho Lazare Sika, Emily D Carter, Rosine Mosso, Abdul Dosso, Alejandra Leyton, Shane M Khan

**Affiliations:** 1 Institute for International Programs, Johns Hopkins Bloomberg School of Public Health, Baltimore, Maryland, USA; 2 Department of Global Community Health and Behavioral Sciences, Tulane University School of Public Health and Tropical Medicine, Tulane, New Orleans, Louisiana, USA; 3 Ecole Nationale Supérieure de Statistique et d’Economie Appliquée, Abidjan, Côte d’Ivoire; 4 Johns Hopkins Center for Communication Programs, Abidjan, Côte d’Ivoire; 5 Division of Data, Research and Policy, UNICEF, New York, New York, USA

## Abstract

**Background:**

Population-based measures of intervention coverage are used in low- and middle-income countries for program planning, prioritization, and evaluation. There is increased interest in effective coverage, which integrates information about service quality or health outcomes. Approaches proposed for quality-adjusted effective coverage include linking data on need and service contact from population-based surveys with data on service quality from health facility surveys. However, there is limited evidence about the validity of different linking methods for effective coverage estimation.

**Methods:**

We collaborated with the 2016 Côte d’Ivoire Multiple Indicator Cluster Survey (MICS) to link data from a health provider assessment to care-seeking data collected by the MICS in the Savanes region of Côte d’Ivoire. The provider assessment was conducted in a census of public and non-public health facilities and pharmacies in Savanes in May-June 2016. We also included community health workers managing sick children who served the clusters sampled for the MICS. The provider assessment collected information on structural and process quality for antenatal care, delivery and immediate newborn care, postnatal care, and sick child care. We linked the MICS and provider data using exact-match and ecological linking methods, including aggregate linking and geolinking methods. We compared the results obtained from exact-match and ecological methods.

**Results:**

We linked 731 of 786 care-seeking episodes (93%) from the MICS to a structural quality score for the provider named by the respondent. Effective coverage estimates computed using exact-match methods were 13%-63% lower than the care-seeking estimates from the MICS. Absolute differences between exact match and ecological linking methods were ±7 percentage points for all ecological methods. Incorporating adjustments for provider category and weighting by service-specific utilization into the ecological methods generally resulted in better agreement between ecological and exact match estimates.

**Conclusions:**

Ecological linking may be a feasible and valid approach for estimating quality-adjusted effective coverage when a census of providers is used. Adjusting for provider type and caseload may improve agreement with exact match results. There remain methodological questions to be addressed to develop guidance on using linking methods for estimating quality-adjusted effective coverage, including the effect of facility sampling and time displacement.

Population-based intervention coverage data are used globally and in low- and middle-income country (LMIC) settings for prioritization, planning, and evaluation purposes. Intervention coverage is defined as the proportion of individuals in need of an intervention who receive it, and these data are primarily measured through household surveys such as the Demographic and Health Surveys (DHS) and the Multiple Indicator Cluster Surveys (MICS) [1,2]. Although these surveys provide valuable national and regional population-based estimates, with great efforts to limit non-sampling error, there are limitations to what they can measure. Respondents may be unable to report on whether they received certain interventions, their need for the intervention, and/or service quality [3]. Surveys of health facilities like the Service Provision Assessments (SPA) [4] and the Service Availability and Readiness Assessments (SARA) [5] seek to measure facility readiness to provide services or the quality of services provided. However these assessments, while important tools for measuring readiness and quality of care, cannot provide population-based estimates.

There is increasing interest in developing measures of “effective coverage” that aim to estimate the proportion of the population in need that receive a health intervention at a level of quality necessary to derive a health benefit [6]. In some cases, this is done by incorporating measures of service quality, or health benefit gained from an intervention, into household surveys [7]. A number of recent studies have combined, or “linked”, data from independently-sampled household and health facility surveys in various ways to generate measures of effective coverage [8-11]. This approach raises several methodological issues about how to combine these estimates, outlined by Do et al [3].

In low-income countries and some middle-income countries, the ideal method for combining household and health provider data is at the individual level, or exact-match linking, where information for each care-seeking episode (ie, every time an individual in need of care seeks care) recorded in a household survey is linked to information about the quality of care of the specific provider(s) visited during that episode (In this paper we will use the term “health provider” or “provider” to encompass both health facilities and non-facility sources of care such as community health workers and pharmacies). However, this approach is not considered feasible for routine national household surveys in most low-income countries because of the difficulty in obtaining an accurate and comprehensive list of providers that can be integrated into household surveys, the challenges in asking respondents about where they sought care, and the resources required to collect data on all providers potentially visited [3]). In middle- and high-income countries with high quality individual medical records, other approaches may be possible. Another approach, ecological linking, involves linking each care-seeking episode in a household survey to an average quality of care score of providers within certain administrative or geographical boundaries of the household survey cluster, or the quality score of the nearest provider(s) (not necessarily the provider visited by the respondent) [12].

As this is a relatively new area of research, little is known about the validity of different ecological linking methods for effective coverage estimation. Similarly, there is limited published research on the impact on effective coverage measures of including non-facility or non-public providers – important sources of care in some settings – in the provider survey sampling frame [8]. Two previous studies in this Collection have compared exact-match and ecological linking methods for delivery care and sick child care to assess the validity of ecological linking methods for coverage estimation [13,14]. Willey et al. found that in one district of Uganda, ecological linking methods that accounted for provider type resulted in better agreement with exact match linking methods, relative to ecological linking only by administrative area [14]. Carter et al. concluded that in five health facility catchment areas in Southern Zambia, excluding community health workers (CHWs) from a provider survey could result in an underestimation of effective coverage [15].

To better understand the feasibility and comparability of exact-match and different ecological methods for linking household and health provider surveys to obtain effective coverage measures, the Improving Coverage Measurement (ICM) project collaborated with the 2016 Côte d’Ivoire MICS to conduct a study of linking methods in the Savanes region of Côte d’Ivoire. This paper presents the effective coverage measures obtained using exact match and different ecological linking methods for maternal, newborn, and child health services, assesses the agreement between these approaches, and discusses the feasibility of these methods.

## METHODS

### Setting

Côte d’Ivoire was selected for this study because of the willingness of the MICS team to collaborate on the study, and because of the timing of the MICS in Côte d’Ivoire. The study was conceptualized in 2015, and the timing of the 2016 Côte d’Ivoire MICS allowed us to conduct the provider assessment concurrently with the MICS, such that we were able to measure quality of care roughly around the time that actual care was sought for sick children.

The Savanes region of Côte d’Ivoire (which includes the health regions of Poro, Tchologo, and Bagoué) has a population of approximately 1.6 million people, 30% of whom live in urban areas [16]. The region includes 5 health districts (Boundiali, Ferkessedougou, Korhogo, Ouangolodougou, Tengrela). The landscape of allopathic providers is dominated by public first-level health centers, but the region also includes several public referral facilities, NGO and religiously-affiliated health facilities, and, in urban areas, private pharmacies and clinics. In addition, some CHWs in the region have been trained to provide treatment for conditions such as malaria. This region was chosen for the study in part because the MICS survey domain (Nord) overlapped exactly with the boundaries of administrative and health regions (ie, the administrative region and health regions were not split between two survey domains), which greatly simplified the development of the sampling frame for the health provider assessment. In addition, the region is of interest to the Ministry of Health and partners because of its below-average indicators.

### Household survey

The 2016 Côte d’Ivoire MICS (MICS-CI) was conducted independently by UNICEF and the National Institute of Statistics in Côte d’Ivoire. A detailed description of the MICS-CI is available elsewhere [17]. Briefly, 512 census enumeration areas (EAs) were sampled, including 44 in the Savanes region (Nord survey domain). Each sampled EA was mapped and its households enumerated. From this list of households, 25 households were sampled in each enumeration area. In each sampled household that consented to participate, a household listing was completed, and interviews were conducted with women aged 15 to 49 years, and with mothers or caregivers of children aged less than 5 years. The fieldwork in the Nord region was conducted from May 12 to July 15, 2016.

The core MICS5 questionnaires were adapted for use in Côte d’Ivoire. Women with a live birth in the 2 years preceding the survey were asked about their antenatal care (ANC) attendance during the pregnancy, where they gave birth, and whether, where, and when they and their newborn received health checks after birth. Mothers and caregivers of children aged less than 5 years were asked whether their child had fever, cough, or diarrhea in the two weeks before the survey, and, if yes, whether and where advice or treatment was sought. For this study, six questions were added to the MICS to identify exact sources of care. Any respondent who reported seeking antenatal care, delivery care, postnatal/postpartum care, or sick child care (for fever/cough or diarrhea) was asked the name(s) of the health provider(s) from whom care was sought. Interviewers received an additional half day of training on these questions, including how to probe for health provider names and how to handle cases where the name of the provider was unknown.

To standardize the coding of health provider names, prior to the MICS fieldwork, study investigators worked with the Ministry of Health, UNICEF, and the national association of pharmacists to obtain lists of health facilities, pharmacies, and community health workers in the Savanes region. Public first-level and referral facilities; private clinics and polyclinics; religious and NGO clinics and hospitals; and pharmacies and pharmacy depots were included. Shops, informal drug sellers, and traditional practitioners were excluded. In addition, community health workers (CHWs) serving any of the 44 sampled EAs in the Savanes region and providing case management for sick children were included. Study investigators and a representative from the Ministry of Health conducted a mission to the Savanes region to meet with local health authorities and health providers in order to identify providers missing from the list, and to remove from the list any providers that were closed or otherwise not providing health services. The resulting list of providers (including CHWs, who were listed by their name) was pre-coded and integrated into the MICS questionnaires to serve as the response options for questions asking about the name of the health provider consulted. The MICS questionnaires also included an “other” response option if the provider named by the respondent was not pre-coded. Interviewers were asked to specify the name(s) of non-pre-coded providers and these were reviewed during data cleaning and recoded if necessary. This list also identified the providers to be included in the health provider assessment, as described below.

The MICS provided cluster geodata for the Nord region collected during the mapping. The cluster geocodes were not displaced, and represented the approximate center of the cluster, or, in some cases, a major landmark in the cluster.

### Health provider assessment

An assessment was conducted in a census of all public first-level and referral health facilities; private clinics and polyclinics; NGO and religiously-affiliated clinics and hospitals; and pharmacies and pharmacy depots in the Savanes region in May-June 2016. In addition, CHWs serving any of the 44 sampled EAs in the Savanes region and providing case management for sick children were included.

As described above, a comprehensive list of health providers was developed in advance of the assessment. In addition, while data collectors were in the field, they identified 40 health providers that had not been included on the original list, and included them in the data collection.

The Service Provision Assessment (SPA) and the Service Availability and Readiness Assessment (SARA) questionnaires were adapted for this provider assessment [4,5]. A facility inventory included questions about general infrastructure and equipment, laboratory services, drugs and diagnostics, and ANC services, delivery care and postnatal care services, and child health services. In addition, service-specific caseload data were abstracted from registers during facility visits. The inventory was administered to the facility in-charge, or to another health worker able to respond to the questions if the in-charge was absent. Data collectors also conducted a listing of health workers at the facility and the types of services that they provided. Health workers providing ANC, delivery or postnatal care, or child health services were interviewed about their training and supervision. For health workers who said that they assisted with deliveries, a module provided by the Informed Decisions for Actions in Maternal and Newborn Health (IDEAS) project was administered to ask about the interventions provided during the last delivery they assisted within the 12 months before the survey [7].

For each health facility and CHW, data collectors aimed to observe two postnatal consultations occurring within seven days of birth and two sick child consultations. SPA observation protocols were adapted for the sick child consultations, and a new observation protocol was developed for routine postnatal consultations. Exit interviews with the woman or mother/caregiver were conducted after each observation. Women and mothers or caregivers were consented for the observation and exit interview prior to the consultation. Newborns brought to the provider because they were ill (rather than for a routine postnatal consultation) were ineligible for the postnatal observation, but were considered for the sick child observation if they met other eligibility criteria. Sick children aged less than 5 years who were brought to the health provider for any of the following signs were eligible to participate: danger signs (change in consciousness/lethargy, convulsions, vomiting everything, not eating or drinking), fever or malaria, cough, fast or difficult breathing, diarrhea or vomiting, ear problem, measles, or nutrition or feeding problems. Children with danger signs who were immediately hospitalized or referred to another provider were excluded.

Data collectors were university students or graduates in a social science or health field who were fluent in a local language (Dioula and Senoufo). Each team also had at least one data collector with clinical training, generally a nurse or midwife. Data collectors received two weeks of training in April 2016 in Abidjan, including role plays and practice observations during a pilot survey in health facilities near and in Abidjan. Data collectors were supervised by study investigators; the Ministry of Health also participated in a supervision mission.

Data were collected on Acer tablets using the CSPro mobile application. Completed forms were uploaded to a secure Box server nightly. Data were cleaned and analyzed in Stata versions 13, 14, and 15 (Stata Inc, College Station, TX, USA) and QGIS version 12.8 (Open Source Geospatial Foundation Project, Beaverton, OR, USA).

### Ethics

The health provider assessment and linking analysis were reviewed by the Johns Hopkins School of Public Health Institutional Review Board-X and the Côte d’Ivoire National Ethics Committee for Research (CNER) at the Ministry of Health and Public Hygiene. Informed consent was sought from health providers, health workers interviewed, and women and caregivers of children whose consultations were observed. De-identified MICS data sets were used for the linking analysis.

### Analysis

#### Care-seeking measures

Using the de-identified MICS-CI data sets for the Nord region, we created care-seeking variables (coded as 1/0) for antenatal care attendance (at least one consultation; at least 4 consultations); delivery in a health facility; post-discharge postnatal consultation within two days of birth for the baby; post-discharge postnatal consultation within 2 days of birth for the mother; and care-seeking for children with fever, signs of acute respiratory infection (rapid/difficult breathing due to a problem in the chest), or diarrhea in the 2 weeks before the survey. Women who reported seeking care for themselves or their children from a traditional healer, traditional birth attendant, informal drug seller, shop, or market were coded as 0 (no care-seeking), as these are considered unskilled providers who are unlikely to provide appropriate case management.

For each of these care-seeking variables except ANC, we recoded the MICS-CI variables about type of provider as public first-level; public referral; non-public clinic; pharmacy (for sick child care); or CHW (for sick child care). The MICS questions about provider type did not distinguish between private, NGO, and religious facilities, and the number of women seeking care for themselves or their children from these facilities was very small (**Table 1**). MICS does not ask about the type of facility visited for ANC.

**Table 1:** Coverage of care-seeking and sources of care, 2016 Côte d’Ivoire MICS

	n/N*	%	95% CI
**ANC1**	321/392	81.9	71.3-89.2
**ANC4**	125/392	31.9	24.6-40.1
**Delivery:**	255/392	65.2	53.2-75.5
-Public referral	51	13.1	8.4-19.7
-Public 1st level	174	44.5	35.6-53.7
-Other public	7	1.9	0.7-5.1
-Private maternity ward	2	0.6	0.1-2.5
-Private clinic	19	4.8	2.1-10.8
-Other private	1	0.3	0.1-2.5
**Post-discharge postnatal care for babies within 2 d of birth:**	25/392	6.4	3.2-12.5
-Public referral	2	0.6	0.2-2.5
-Public 1st level	18	4.5	2.1-9.4
-Private clinic	3	0.7	0.2-2.9
-Other	2	0.6	0.1-2.5
**Post-discharge postnatal care for mothers within 2 d of birth:**	20/392	5.1	2.4-10.7
-Public referral	4	1.0	0.3-3.1
-Public 1st level	10	2.4	1.1-5.2
-Private clinic	5	1.3	0.4-4.3
-Other	1	0.4	0.1-2.7
**Care-seeking for children 0-59 mo with diarrhea, fever or signs of acute respiratory infection:†**	79/183	43.2	35.7-60.0
-Public referral	13	7.1	3.0-15.9
-Public 1st level	48	27.0	19.7-36.0
-Private clinic	2	1.1	0.2-7.7
-CHW	1	0.2	0.03-1.8
-Pharmacy	15	8.2	4.6-14.2

MICS – Multiple Indicator Cluster Survey, CI – confidence interval, CHW – community health worker

*Weighted n/N.

†Rapid/difficult breathing due to a problem in the chest.

#### Quality of care

We used the definitions established by Donabedian for structural quality and process quality [18]. We created indices of provider structural quality and process quality from the health provider assessment data for each of the following services: ANC; labor and delivery; immediate newborn care; routine postnatal consultations; and sick child management. Structural quality (ie, readiness) refers to the service environment of a provider, including the material and human resource attributes of the provider, while process quality refers to the quality of the actual processes of care, including the patient’s activities, the provider’s activities, and the interactions between the two.

For each type of service, using the SARA analysis guide, we identified items of structural quality in the domains of service availability; availability of drugs, diagnostics, and commodities; and training, supervision, and availability of guidelines. We did the same for process quality using the WHO/UNICEF guidelines for labor & delivery, immediate newborn care, postnatal care, and integrated management of childhood illness [19-21]. Each item was coded as 1 (present) or 0 (not present), except for training variables, for which we used the proportion of health workers at the facility who had received the training. For each service, the items were summed and divided by the total number of elements to produce a structural quality score that could range from 0 to 1. Thus, all items contributed equally to the score. The components of these indices are listed in Table S1 and Table S2 in **Online Supplementary Document(Online Supplementary Document)**.

At pharmacies we only collected data on the availability of drugs and commodities; we assigned pharmacies a score of 0 for all service availability, training, and supervision variables. CHWs were treated like health facilities in that we collected data on service availability, training, supervision, and availability of drugs and commodities, and their scores comprised all of these domains.

#### Exact-match linking analysis

In the exact-match analysis, each care-seeking episode in the MICS-CI data sets was assigned the process and structural quality scores for the specific health provider(s) visited during the care-seeking episode. If a woman or child visited more than one provider, the scores of those providers were averaged. If no score was available for a provider, we imputed an average score based on the managing authority and level of the provider.

#### Ecological linking analysis

To link household and provider surveys using an ecological analysis, care-seeking variables from the household survey are either assigned an average quality of care score computed across a set of providers, or are assigned the score of the nearest provider. The average or nearest quality score can be calculated in a number of ways, including an average quality of care score for an administrative area and/or provider category, the quality score of the closest provider (straight-line distance or road distance), or an average score for all providers within a certain radius of the household or cluster [12]. We conducted five types of ecological analyses, as described below.

#### Aggregate ecological analyses

**By district:** For each individual who sought care, we assigned the average structural and process quality scores computed across all providers in the district where the household survey cluster was located.

**By district and provider category:** For each individual who sought care, we assigned the average structural and process quality scores for the provider category reported as the source of care (public first level facility, public referral facility, private first level, private referral, pharmacy (child care-seeking only), and CHW (child care-seeking only)), within the district where the household survey cluster was located. This type of linking was not performed for ANC, since the MICS-CI did not ask women what type of facility they attended for ANC.

**By distance (Euclidean buffer):** For each individual who sought care, we assigned the average structural and process quality scores, computed across all providers within a 10 km radius of the central point of the household survey cluster. If there was no provider within a 10 km radius, the individual was linked to the nearest provider by straight-line distance.

#### Single-provider ecological analyses

**Straight-line distance:** For each individual who sought care, we assigned them the structural and process quality scores of the provider nearest the survey cluster, with distance calculated as a straight line from the central point of the household survey cluster.

**Road network distance:** For each individual who sought care, we assigned them the structural and process quality scores of the provider nearest the survey cluster, with distance calculated as the distance over the road network from the central point of the household survey cluster.

The categorization of providers was based solely on the MICS5 questions regarding the type of provider seen; we did not use the additional information collected on provider names to correct this categorization, as we wanted to simulate what would typically be available from a household survey for ecological linking.

For single-provider methods, both straight-line and road distance, we compared two different approaches: 1) linking women and children to the nearest provider, and 2) linking women and children to the nearest provider within the same category that they reported visiting in the MICS-CI. For example, if a woman reported giving birth at a public first level facility, she was linked first to the nearest provider of any kind, and then to the nearest public first level facility.

We did not adjust for provider category for the 10km buffer method, as in many cases this would have resulted in linking to a single provider (or no provider).

To better reflect the quality of facilities that are used by the population, we computed weighted provider averages for all ecological linking methods that were based on an average of provider scores (by district, by district and provider category, and Euclidean buffer). We weighted individual provider scores by the service-specific caseload of the provider (eg, number of ANC consultations in the 3 months prior to the assessment for ANC scores; number of deliveries in the 3 months prior to the assessment for delivery scores). We report linking results using both the weighted and unweighted averages.

Finally, to assess the effect of including only public sector facilities in a health provider assessment, we limited the provider data set to only public sector facilities and re-ran all of the ecological linking methods described above using this limited data set.

#### Effective coverage estimates

For each of the linking methods described above, we calculated structure- and process-adjusted measures of coverage as, respectively, the mean of the structural or process quality scores for all women and children needing care. Women and children who needed care but did not seek it (for example, women who did not attend ANC during their pregnancy, or sick children who were not taken for care) were assigned a score of 0, as were women and children who sought care from traditional healers, traditional birth attendants, shops, or informal drug sellers. MICS-CI survey weights were used for all analyses, and standard errors were calculated using the Taylor linearization method. Because we conducted a census of health facilities and non-facility providers, we assumed that there was no sampling error associated with the facility quality estimates.

We examined the absolute and relative differences between ecological and exact match estimates for each type of service and used Wald tests to assess whether differences between the estimates were statistically significant.

## RESULTS

### Household survey

The MICS-CI interviewed 1007 households out of 1100 sampled in the Nord region of Côte d’Ivoire, including 1077 women 15-49 years and 939 children under 5 years (weighted numbers). 36% of women (392/1077 women) delivered a child within two years before the survey. Nine percent of children under five were reported to have had diarrhea in the past two weeks, 15% had fever, and 0.2% had signs of acute respiratory infection (rapid or difficult breathing due to a problem in the chest). Coverage of care-seeking for antenatal, delivery, postnatal, and sick child care, along with reported sources of care, are shown in **Table 1**. Care-seeking was lowest for postnatal visits within 2 days for babies (6.4%) and mothers (5.1%), and highest for deliveries (65.2%) and at least one antenatal visit (81.9%). Across services, most care-seeking took place in public first-level facilities. However, the informal sector was also a relatively important source of care for sick children, with 21 care-seeking episodes (of 183 sick children) from informal drug vendors, 10 from traditional healers, and one from a shop (data not shown). Care-seeking from multiple providers was very rare: only one child was reported to have been taken to more than one provider in the formal sector (public first level and referral facilities).

### Health provider assessment

Of the 350 health facilities, pharmacies, and CHWs listed in the sampling frame, 75 (21%) were not found, were not operational, were determined to be ineligible (did not provide antenatal, delivery, postnatal, or sick child care for children under 5), or did not consent to participate. These included 27 private facilities, 22 public facilities, 16 CHWs, and 10 pharmacies and depots. Forty new eligible providers were found during data collection, including 23 CHWs, 6 private or religious facilities, and 2 public facilities. In total, the health provider assessment collected data from 194 health facilities, 43 CHWs, and 78 pharmacies or pharmacy depots in the Nord region. The distribution of health providers by level and managing authority is shown in **Table 2**. We interviewed 633 health workers at these providers and observed 183 routine postnatal consultations and 344 sick child consultations.

**Table 2:** Level and managing authority of health facilities, pharmacies, and CHWs included in the assessment

Facility type	Overall	Rural	Urban
**Public:**			
Referral	5	0	5
1st level	144	97	47
CHW	43	31	12
**Religious:**			
Referral	2	0	2
1st level	11	3	8
**Private/non-profit:**			
Clinic	32	2	30
Pharmacy/depot	78	29	49
**Total**	315	162	153

The distribution of structural and process quality scores by provider type and type of service is shown in **Figure 1** and **Figure 2**. The distribution of scores by district and urban/rural localization is shown in l Figures S1-S10 in **Online Supplementary Document(Online Supplementary Document)**. The median caseload is shown by provider and service type in Table S3 in **Online Supplementary Document(Online Supplementary Document).**

**Figure 1:**
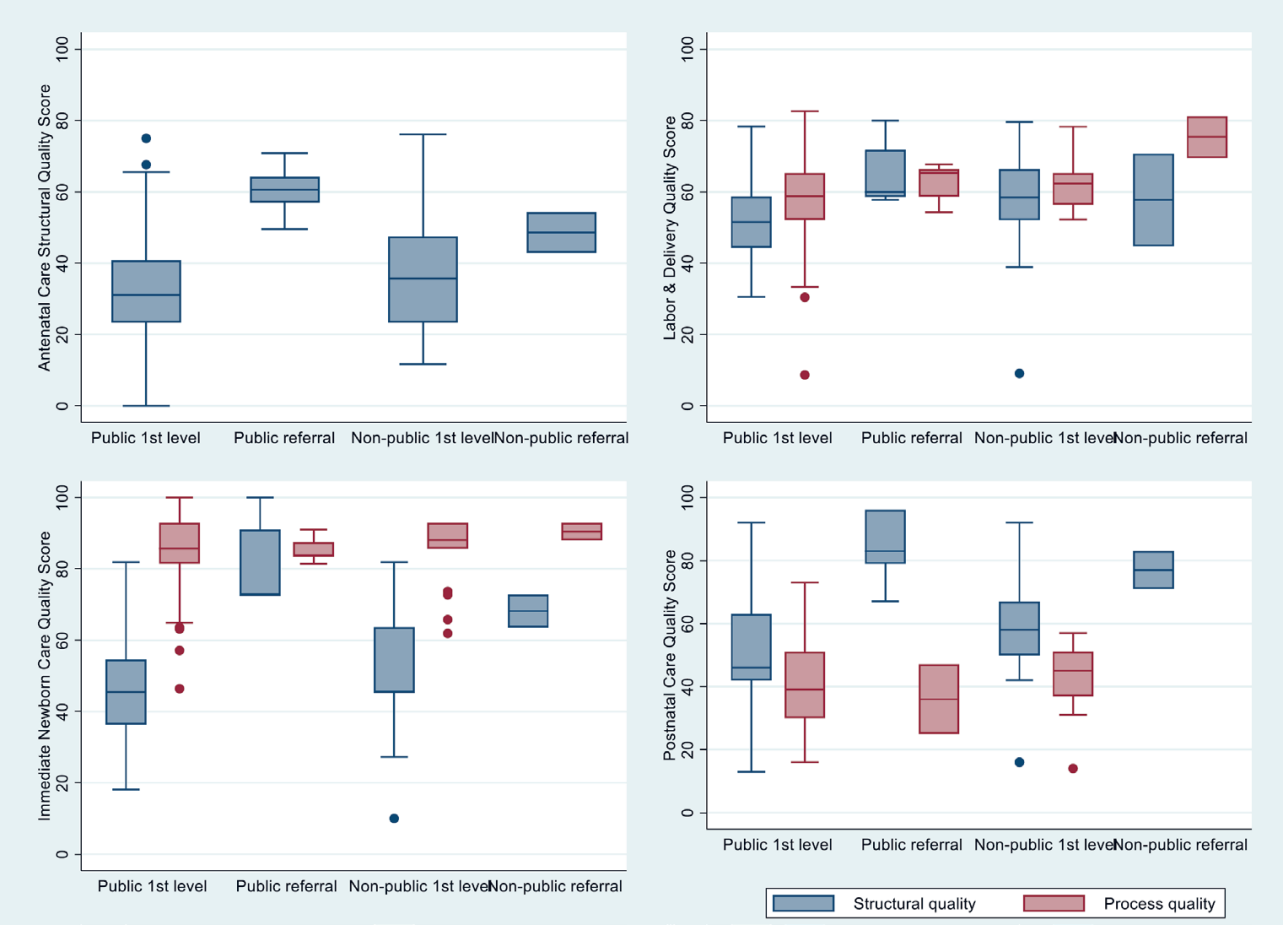
Distribution of antenatal, labor & delivery, immediate newborn, and postnatal care (PNC) quality scores by facility type.

**Figure 2:**
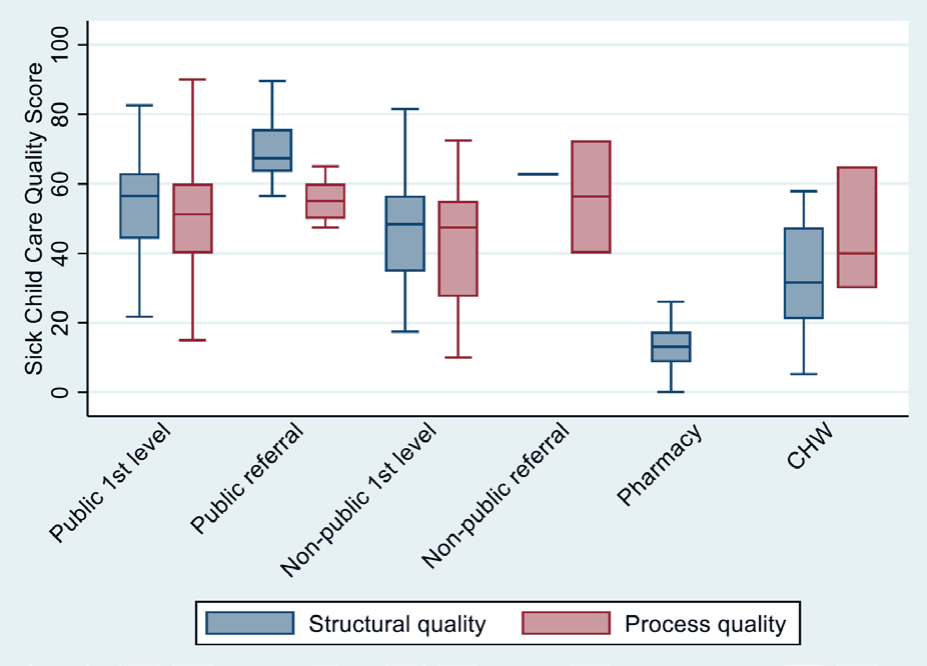
Distribution of sick child care quality scores by facility type.

### Exact-match linking

For exact-match linking, we were able to link 731 of 786 care-seeking episodes (93%) from the MICS to a structural quality score for the provider named by the respondent. Exact-match linking rates were similar for ANC (93.7%), delivery (92.3%), postnatal care for women (94.7%), and sick child (93.9%) care-seeking episodes, and somewhat lower for postnatal care for babies (88.1%). We were able to impute scores for the 55 unmatched episodes based on the average score for the reported provider category in the district of residence of the respondent. Reasons for unmatched care-seeking episodes were similar across types of care-seeking and included 1) the provider visited by the respondent was not included in the provider assessment because it was located outside the study region, refused to participate, was closed/not operational, or was included but reported that they did not provide the service (n = 25), 2) the CHW post name (village name) rather than the CHW name was recorded as the source of care in the household data (n = 7), 3) error in recording source of care in the household data such that it was unusable (n = 9), 4) source of care was reported as “don’t know” in the household data (n = 3), and 5) source of care was missing in the household data (n = 11).

### Effective coverage

Effective coverage estimates calculated using exact-match and ecological linking are reported in **Tables 3 to 8** by type of service. Effective coverage estimates based on exact-match methods were 13%-63% lower than the care-seeking measures from the MICS. Process-adjusted measures for sick child care and postnatal care, for which we had process quality scores based on observation of delivered care, were lower than structure-adjusted measures. Process-adjusted measures were higher than structure-adjusted measures for labor & delivery and immediate newborn care, for which we relied on provider reports of the interventions delivered.

**Table 3:** Coverage estimates for antenatal care (N = 392)

	At least 1 ANC				At least 4 ANC			
	**%**	**95% CI**	**Absolute difference**	**Relative difference**	**%**	**95% CI**	**Absolute difference**	**Relative difference**
Antenatal care attendance (not adjusted for service quality)	81.9	71.3-89.2			31.9	24.6-40.1		
**Structure-adjusted coverage – exact match method**								
Exact match	30.9	27.1-34.8	REF	REF	12.1	8.9-15.3	REF	REF
**Structure-adjusted coverage – aggregate methods**								
Aggregate by district unweighted	28.4*	25.7-31.1	-2.5	-8.1%	10.8	8.3-13.3	-1.3	-10.7%
Aggregate by district weighted	34.2*	30.8-37.6	3.3	10.7%	13.2	10.0-16.3	1.1	9.1%
10 km buffer unweighted	28.1	24.2-32.0	-2.8	-9.1%	10.8	8.2-13.4	-1.3	-10.7%
10 km buffer weighted	30.6	26.4-34.7	-0.3	-1.0%	12.0	9.1-15.0	-0.1	-0.8%
**Structure-adjusted coverage – single-facility methods**								
Nearest facility (straight line)	29.6	24.9-34.2	-1.3	-4.2%	11.7	8.2-15.1	-0.4	-3.3%
Nearest facility (road distance)	28.1*	23.8-32.4	-2.8	-9.1%	10.8	8.0-13.6	-1.3	-10.7%

ANC – antenatal care, CI – confidence interval

*Exact-match and ecological estimates are significantly different at the *P* < 0.05 level.

**Table 4:** Coverage estimates for labor & delivery (N = 392)

	%	95% CI	Absolute difference	Relative difference	%	95% CI	Absolute difference	Relative difference	
Facility delivery (not adjusted for service quality)	65.2	53.2-75.5							
	**Structure-adjusted coverage**				**Process-adjusted coverage**			
**Linked coverage estimates – exact match method**								
Exact match	37.2	30.5-43.9	REF	REF	40.1	32.9-47.3	REF	REF	
**Linked coverage estimates – aggregate methods**								
Aggregate by district (unweighted)	34.9*	29.0-40.8	-2.3	-6.2%	39.0	32.3-45.7	-1.1	-2.7%	
Aggregate by district (weighted)	37.9	31.3-44.4	0.7	1.9%	40.7	33.7-47.7	0.6	1.5%	
Aggregate by district and provider category (unweighted)	37.0	30.4-43.6	-0.2	-0.5%	39.7	32.7-46.7	-0.4	-1.0%	
Aggregate by district and provider category (weighted)	38.8*	31.9-45.7	1.6	4.3%	40.8	33.6-48.0	0.7	1.7%	
10 km buffer (unweighted)	35.3*	29.3-41.4	-1.9	-5.1%	39.1	32.0-46.2	-1.0	-2.5%	
10 km buffer (weighted)	37.5	30.6-44.4	0.3	0.8%	39.8	32.5-47.1	-0.3	-0.7%	
**Linked coverage estimates – single-facility methods**								
Nearest facility (straight line)	36.5	29.5-43.5	-0.7	-1.9%	39.8	32.2-47.5	-0.3	-0.7%	
Nearest facility (straight line) by provider category	37.0	30.0-44.0	-0.2	-0.5%	39.6	32.1-47.1	-0.5	-1.2%	
Nearest facility (road distance)	36.8	29.8-43.9	-0.4	-1.1%	40.4	32.6-48.1	0.3	0.7%	
Nearest facility (road distance) by provider category	37.5	30.4-44.6	0.3	0.8%	40.2	32.5-47.9	0.1	0.2%	

CI – confidence interval

*Exact-match and ecological estimates are significantly different at the *P* < 0.05 level.

**Table 5:** Coverage estimates for immediate newborn care (N = 392)

	%	95% CI	Absolute difference	Relative difference	%	95% CI	Absolute difference	Relative difference
Newborns born in a facility (not adjusted for service quality)	65.2	53.2-75.5						
	**Structure-adjusted coverage**				**Process-adjusted coverage**			
**Linked coverage estimates – exact match method**								
Exact match	36.1	28.7-43.6	REF	REF	56.5	46.6-66.3	REF	REF
**Linked coverage estimates – aggregate methods**								
Aggregate by district (unweighted)	31.9	26.4-37.3	-4.2*	-11.6%	56.0	46.2-65.7	-0.5	-0.9%
Aggregate by district (weighted)	37.4	30.9-43.9	1.3	3.6%	55.9	46.1-65.7	-0.6	-1.1%
Aggregate by district and provider category (unweighted)	37.6	30.0-45.2	1.5	4.2%	55.6*	46.0-65.3	-0.9	-1.6%
Aggregate by district and provider category (weighted)	39.7	31.8-47.6	3.6	10.0%	55.7	46.0-65.4	-0.8	-1.4%
10 km buffer (unweighted)	31.5*	25.8-37.1	-4.6	-12.7%	56.3	46.3-66.3	-0.2	-0.4%
10 km buffer (weighted)	36.1	28.7-43.4	0	0.0%	56.5	46.5-66.6	0	0.0%
**Linked coverage estimates – single-facility methods**								
Nearest facility (straight line)	32.5*	25.2-39.8	-3.6	-10.0%	55.6	45.6-65.5	-0.9	-1.6%
Nearest facility (straight line) by provider category	37.4	29.1-45.7	1.3	3.6%	55.5	45.7-65.3	-1	-1.8%
Nearest facility (road distance)	33.7	26.2-41.2	-2.4	-6.6%	55.6	45.565.8	-0.9	-1.6%
Nearest facility (road distance) by provider category	38.6	30.2-47.0	2.5	6.9%	55.8	45.8-65.8	-0.7	-1.2%

CI – confidence interval

*Exact-match and ecological estimates are significantly different at the *P* < 0.05 level.

**Table 6:** Coverage estimates for routine postnatal consultations for newborns (N = 392)

	%	95% CI	Absolute difference	Relative difference	%	95% CI	Absolute difference	Relative difference
Received a post-discharge PNC consultation within 2 d of birth (not adjusted for service quality)	6.4	3.2-12.5						
	**Structure-adjusted coverage**				**Process-adjusted coverage**			
**Linked coverage estimates – exact match method**								
Exact match	3.8	1.1-6.6	REF	REF	2.7	0.8-4.6	REF	REF
**Linked coverage estimates – aggregate methods**								
Aggregate by district (unweighted)	3.8	1.1-6.6	REF	REF	2.7	0.8-4.6	REF	REF
Aggregate by district (weighted)	3.4	1.1-5.7	-0.4	-10.5%	2.6	0.8-4.3	-0.1	-3.7%
Aggregate by district and provider category (unweighted)	3.9	1.2-6.6	0.1	2.6%	2.8	0.9-4.7	0.1	3.7%
Aggregate by district and provider category (weighted)	3.5	1.2-5.8	-0.3	-7.9%	2.5	0.7-4.2	-0.2	-7.4%
10km buffer (unweighted)	3.3	1.0-5.7	-0.5	-13.2%	2.7	0.7-4.7	0	0.0%
10 km buffer (weighted)	3.8	0.9-6.7	0	0.0%	2.8	0.7-4.8	0.1	3.7%
**Linked coverage estimates – single-facility methods**								
Nearest facility (straight line)	3.6	0.9-6.3	-0.2	-5.3%	2.7	0.8-4.6	0	0.0%
Nearest facility (straight line) by provider category	3.6	1.1-6.0	-0.2	-5.3%	2.7*	0.8-4.7	0	0.0%
Nearest facility (road distance)	3.7	1.0-6.0	-0.1	-2.6%	2.7	0.8-4.7	0	0.0%
Nearest facility (road distance) by provider category	3.7	1.2-6.2	-0.1	-2.6%	2.8	0.8-4.8	0.1	3.7%

CI – confidence interval

*Exact-match and ecological estimates are significantly different at the *P* < 0.05 level.

**Table 7:** Coverage estimates for routine postnatal consultations for women (N = 392)

	%	95% CI	Absolute difference	Relative difference	%	95% CI	Absolute difference	Relative difference
Received a post-discharge PNC consultation within 2 d of birth (not adjusted for service quality)	5.1	2.4-10.7						
	**Structure-adjusted coverage**				**Process-adjusted coverage**			
**Linked coverage estimates – exact match method**								
Exact match	3.2	0.6-5.8	REF	REF	2.1	0.4-3.9	REF	REF
**Linked coverage estimates – aggregate methods**								
Aggregate by district (unweighted)	2.6	0.6-4.6	-0.6	-18.8%	2.0	0.5-3.5	-0.1	-4.8%
Aggregate by district (weighted)	3.1	0.7-5.5	-0.1	-3.1%	2.2	0.5-3.9	0.1	4.8%
Aggregate by district and provider category (unweighted)	3.1	0.8-5.4	-0.1	-3.1%	1.7	0.2-3.1	-0.4	-19.0%
Aggregate by district and provider category (weighted)	3.3	0.9-5.8	0.1	3.1%	1.8	0.3-3.3	-0.3	-14.3%
10km buffer (unweighted)	2.7	0.5-4.9	-0.5	-15.6%	2.1	0.4-3.8	0	0.0%
10 km buffer (weighted)	3.2	0.5-5.9	0	0.0%	2.1	0.4-3.8	0	0.0%
**Linked coverage estimates – single-facility methods**								
Nearest facility (straight line)	2.9	0.6-5.2	-0.3	-9.4%	2.0	0.4-3.6	-0.1	-4.8%
Nearest facility (straight line) by provider category	3.1	0.8-5.5	-0.1	-3.1%	1.9*	0.4-3.5	-0.2	-9.5%
Nearest facility (road distance)	2.9	0.6-5.2	-0.3	-9.4%	2.0	0.4-3.6	-0.1	-4.8%
Nearest facility (road distance) by provider category	3.3	0.8-5.8	0.1	3.1%	1.9*	0.3-3.5	-0.2	-9.5%

CI – confidence interval

*Exact-match and ecological estimates are significantly different at the *P* < 0.05 level.

**Table 8:** Coverage estimates for sick child care (N = 183)

	%	95% CI	Absolute difference	Relative difference	%	95% CI	Absolute difference	Relative difference
Care-seeking from a skilled provider for fever, diarrhea, or signs of acute respiratory infection (not adjusted for service quality)	43.2	35.7-60.0						
	**Structure-adjusted coverage**				**Process-adjusted coverage**			
**Linked coverage estimates – exact match method**								
Exact match	22.9	18.2-27.5	REF	REF	16.8	12.8-20.8	REF	REF
**Linked coverage estimates – aggregate methods**								
Aggregate by district (unweighted)	17.8*	14.6-21.0	-5.1	-22.3%	21.0*	17.2-24.8	4.2	25.0%
Aggregate by district (weighted)	19.7*	16.1-23.2	-3.2	-14.0%	21.2*	17.4-25.0	4.4	26.2%
Aggregate by district and provider category (unweighted)	20.3*	15.8-24.8	-2.6	-11.4%	17.4	13.3-21.4	0.6	3.6%
Aggregate by district and provider category (weighted)	21.1*	16.4-25.8	-1.8	-7.9%	17.1	13.1-21.2	0.3	1.8%
10 km buffer (unweighted)	18.8*	14.9-22.7	-4.1	-17.9%	15.7	12.4-19.1	-1.1	-6.5%
10 km buffer (weighted)	19.1*	15.1-23.0	-3.8	-16.6%	15.6	12.1-19.1	-1.2	-7.1%
**Linked coverage estimates – single-facility methods**								
Nearest facility (straight line)	18.2*	12.3-24.1	-4.7	-20.5%	14.3	9.1-19.6	-2.5	-14.9%
Nearest facility (straight line) by provider category	20.8*	16.1-25.4	-2.1	-9.2%	16.5	12.2-20.7	-0.3	-1.8%
Nearest facility (road distance)	16.0*	10.9-21.1	-6.9	-30.1%	13.8	8.5-19.1	-3	-17.9%
Nearest facility (road distance) by provider category	20.2*	16.0-24.4	-2.7	-11.8%	16.5	12.3-21.8	-0.3	-1.8%

CI – confidence interval

*Exact-match and ecological estimates are significantly different at the *P* < 0.05 level.

We assessed the extent to which geo-linking (single provider) methods reproduced actual care-seeking practices and therefore exact-match linking. **Table 9** shows the proportion of individuals who sought care from a skilled provider who were linked to their reported source of care (named provider) using single-provider linking methods. The proportion linked to their exact source of care ranged from 20.3% (road distance, sick child) to 57.1% (straight-line distance by provider category, ANC4); for most methods, fewer than 50% of women/children were linked to their exact source of care. Single-provider methods that accounted for the category of provider visited linked more respondents to their exact source of care, particularly for ANC and sick child care.

**Table 9:** Percentage of individuals who sought care from a skilled provider for whom geo-linking linked to at least one of their exact-match sources of care

Geo-linking method	ANC4	Delivery	PNC	PPC	Sick child
	n = 105	n = 236	n = 16	n = 12	n = 74
Nearest facility (straight line)	43.8%	53.0%	37.5%	33.3%	29.7%
Nearest facility (straight line) by category	57.1%	53.8%	37.5%	33.3%	50.0%
Nearest facility (road distance)	35.2%	46.6%	31.3%	25.0%	20.3%
Nearest facility (road distance) by category	48.6%	48.7%	31.3%	33.3%	43.2%

ANC – antenatal care, PNC – postnatal care for newborns, PPC – postpartum care for mothers

### Comparison of linking methods

Absolute differences between exact match and ecological linking methods were small: ±7 percentage points for all ecological methods and ±4 percentage points for ecological methods that adjusted for provider level. Relative differences were larger and ranged more widely from 0% (postnatal care) to -30.1% (sick child care). Absolute and relative errors were smaller for process-adjusted coverage than for structure-adjusted coverage. The confidence intervals for all ecological estimates overlapped with the confidence intervals for the exact-match estimates, but some ecological estimates were significantly different (*P* < 0.05) from the exact match estimates, particularly the aggregate method by district (unweighted), and all the structure-adjusted estimates for sick child care. We note, though, that even in these cases the differences were often small and not programmatically meaningful.

**Figure 3:**
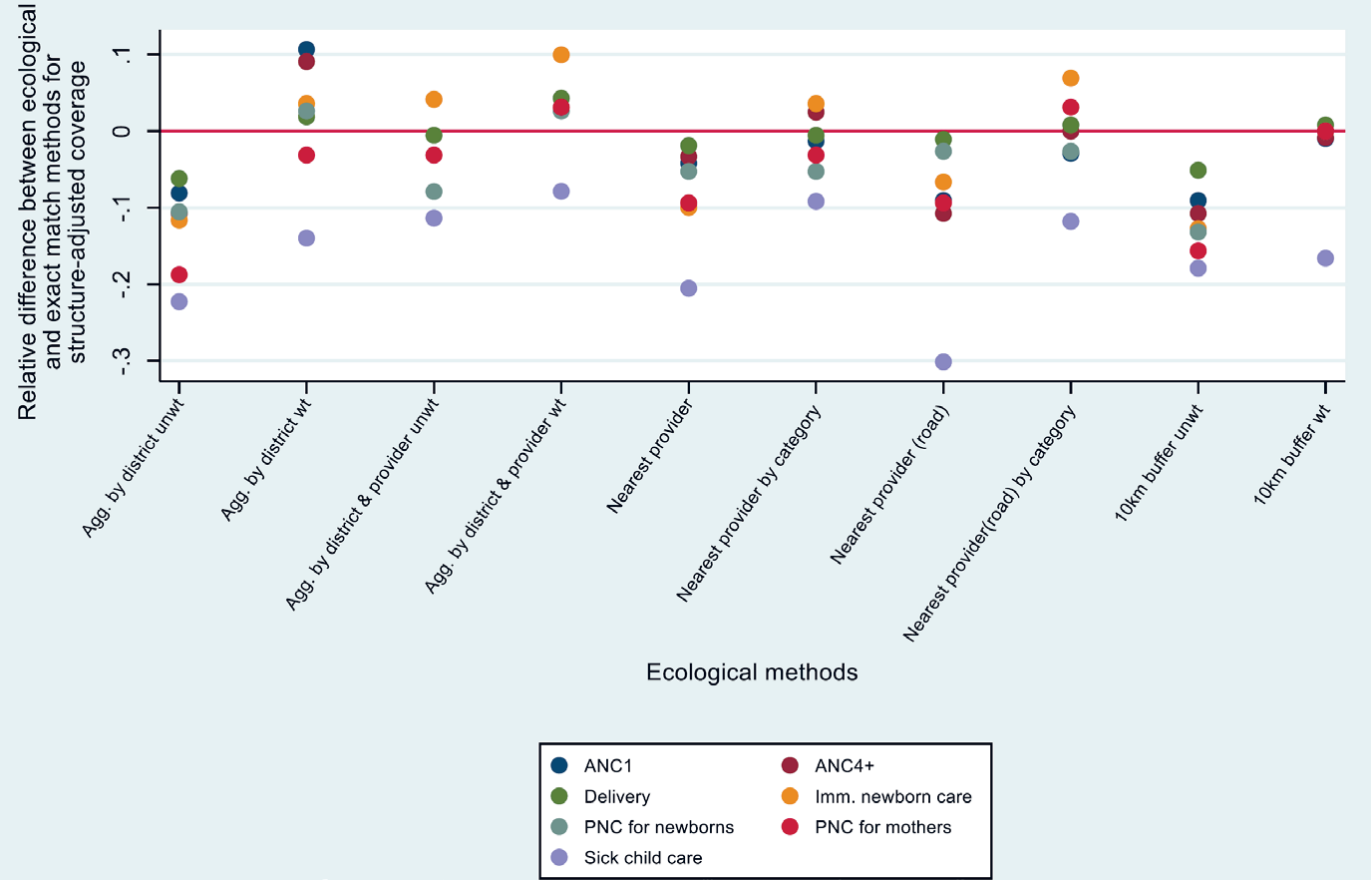
Relative difference in structure-adjusted coverage calculated using ecological linking methods, relative to exact-match linking, by service type.

**Figure 4:**
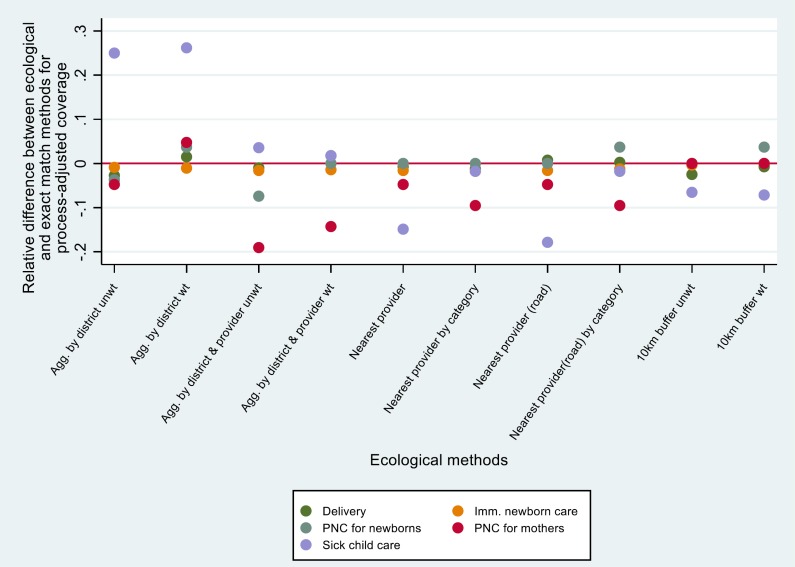
Relative difference between process-adjusted coverage calculated using ecological linking methods and exact-match linking, by service type.

**Figure 3** and **Figure 4** show the relative difference for each ecological estimate (compared to exact-match) by service type. Both aggregate and single-provider ecological methods that were unweighted or did not adjust for facility category tended to underestimate quality-adjusted coverage (negative differences). Weighting by caseload or adjusting for provider category tended to produce differences that were more evenly distributed around 0. Excluding sick child care, the 10km buffer method appeared to produce estimates that were closest to the exact match method for structure-adjusted coverage. For process-adjusted coverage, several methods produced results that were close to exact match estimates, including 10 km buffer (weighted and unweighted), aggregate by district (weighted and unweighted), and road distance. For child health, aggregate by district and provider (weighted) and nearest provider by category produced the best agreement with exact match methods. Again, we note that in many cases the difference between methods was quite small.

The effect of including only public sector facilities in the analysis generally resulted in a similar range of error for all service types except sick child consultations, for which the magnitude of error in the ecological estimates was much smaller when only public sector facilities were included (Table S4 in **Online Supplementary Document(Online Supplementary Document)**).

## DISCUSSION

Linking household and health provider surveys to estimate effective coverage of health interventions may have the potential to improve monitoring and provide more actionable measures of coverage for countries and programs. Like previous studies measuring effective coverage, we found that, using exact matching methods, effective coverage measures of ANC, delivery, newborn, postnatal, and sick child care were significantly lower (by 13 to 63%) than measures of care-seeking calculated from a household survey alone. This is not surprising, as contact with a health provider does not necessarily mean that the necessary interventions were delivered with quality. Other studies have also found large gaps between coverage of care-seeking and effective or quality-adjusted coverage: for example Marchant et al. found in 3 settings that quality coverage of ANC was 82%-93% lower than overall ANC coverage, and quality coverage of delivery was 41%-53% lower than overall coverage of institutional delivery [7]. Leslie et al. found that across 8 countries, effective coverage of ANC was 44% lower than ANC4, and effective coverage of sick child care 62% lower than care-seeking for sick children [10]. These findings highlight the work that remains in ensuring that effective interventions, delivered with high quality, reach individuals in need.

Consensus and guidance on the methods for effective coverage analyses that link household and health provider data are needed. This paper is the third to describe the level of agreement between effective coverage estimates generated using ecological and exact-match linking methods [14,15]. However, this study differs from the preceding two in that in that it used a MICS survey as the source of household data and in that it compared ecological and exact match linking using structure and process quality for maternal, newborn, and child health interventions in one study. We also implemented a number of ecological linking methods using only questions and geographical coordinates that are already collected in standard household and health facility surveys. Studies linking household and health facility surveys for effective coverage estimates have tended to use aggregate linking methods by administrative area [7,22-28]. We contribute to the methodological literature by assessing these and other methods, and by highlighting the improvements in agreement when weighting provider averages and adjusting for provider category.

We found that in this setting there were relatively small differences between ecological and exact match estimates. Although in some cases the estimates were significantly different, the confidence intervals overlapped and the magnitude of the differences was often small enough to be not meaningful from a programmatic standpoint. This is promising as it suggests that ecological linking methods can be used to generate valid measures of effective coverage when exact matching is not feasible. Ecological linking methods are more feasible for implementation than exact match methods, as they do not require modification of household surveys and the data processing and analysis is relatively straightforward (for aggregate ecological linking methods). It is also important to note that some analyses seek to link household and provider surveys in order to study the effect of service environment on care-seeking or intervention coverage, and for those studies it is important that individuals be linked to the specific provider from which they sought care.

The most accurate ecological method (closest to exact match estimate) varied by type of service and whether process or structural quality was used. However, we found that for structure-adjusted measures, the weighted 10km buffer method performed best, except for sick child care, where aggregate methods by district and provider, and road distance by provider category were more accurate. We found that for aggregate ecological methods, adjusting for provider category (level and managing authority) and weighting provider averages by service-specific utilization produced better agreement with exact match estimates, relative to methods that used only an unweighted average of facilities in the administrative area. For single-provider methods, adjusting by provider category also improved agreement. Weighting and adjusting for provider category also tended to result in errors that were more evenly distributed around 0. Weighting by caseload likely reduces the influence of small, low utilization facilities, which may have lower structural and process quality. Adjusting for provider category for single provider linking methods may result in more women and children being linked to their exact source of care. For aggregate linking methods, adjusting for provider category implicitly weights provider categories by utilization.

We caution that our analysis used a census of all providers in the region and undisplaced cluster geo-coordinates, which may have improved the agreement between ecological and exact match linking methods, relative to the use of a sample of providers or displaced cluster data. Future analyses will simulate sampling of health providers and displacement of clusters to assess their effects on ecological estimates.

Our results are largely consistent with those of Willey et al. in Uganda, and they held true over a larger geographic area and for antenatal, postnatal, and sick child care in addition to delivery care [14]. Unlike an earlier study in Zambia, we did not find that including CHWs improved our ecological estimates [15]. However, CHWs were a very infrequent source of care in this setting, whereas they accounted for 18% of care-seeking in rural areas in the Zambia study. Given the challenges in obtaining an accurate sampling frame of CHWs and observing a sufficient number of consultations by CHWs, it is important to consider the role that CHWs play in provision of care when deciding whether to include them in the health provider assessment.

The proportion of women and children linked to their reported source of care using single-provider methods was lower in this study (20%-57%) than in a similar study in Zambia (77%-89%) [15]. Possible reasons for this include provider bypassing, error associated with using cluster centroid location rather than household location (the Zambia study used exact household location), error in recording exact sources of care, or limitations in the geolinking methods. Straight-line geolinking methods do not account for barriers in accessibility (eg, rivers, difficult terrain). Road distance methods attempt to account for accessibility but do not account for road condition. In addition, road network geodata generally do not include footpaths, which are important routes in rural areas. Rural clusters can be very dispersed, with 1km or more between the center of the cluster and outlying households. This dispersion may induce error when calculating the closest facility using the cluster centroid.

We found that the exact match linking method was relatively feasible for implementation alongside a MICS survey at regional level, with 93% of care-seeking episodes linked to the provider visited by the survey respondent. As described in the methods, however, exact match linking required significant work to obtain a list of providers in the study region and incorporate it into the MICS and a large financial investment to collect data from all facilities and pharmacies (and some CHWs) in the region. Even then, the list of providers was imperfect and required updating in the field, which was logistically challenging. In addition, timing the provider assessment to be conducted just before the MICS required significant coordination and flexibility regarding the preparations for the provider assessment. Conducting exact match linking at national level would likely be feasible only if a provider census were already planned for other purposes, and even then would be logistically challenging.

This study had a number of limitations. Although we treated our exact match measure as the “gold standard”, we note that there may have been some degree of error in respondents’ reports of the providers that they visited, as with any survey question. For example, respondents may have mis-reported the provider name, or interviewers could have selected the wrong provider from the list. However, these errors are likely to have been rare and therefore would not have affected our overall results. We also assumed that there was no error in our measurement of facility quality because we collected data from all facilities; this is an anti-conservative assumption that likely would have biased our results towards incorrectly rejecting the null hypothesis that ecological and exact match estimates were not different. However, even with this assumption we found few significant differences between these estimates, except in the case of structure-adjusted sick child care. We note that there is a need for further research on appropriate methods for estimating error when linking household and facility data. In addition, we note that the proportion of women and newborns attending post-discharge PNC visits within 2 days of birth was very low and therefore our linked estimates are based on a small number of cases.

The setting for this study, a primarily rural region of West Africa, has a provider ecosystem that is dominated by the public sector, and to a lesser extent pharmacies, informal drug sellers, and traditional healers. It is thus similar to many other rural sub-Saharan African settings where most care-seeking takers place in the public sector – although we noted above the difference from Zambia with respect to care-seeking from CHWs. However, our results may not be generalizable to urban or South Asian settings where more care-seeking occurs in the private sector. Finally, we had access to undisplaced cluster location data provided by MICS. The DHS programme typically displaces cluster locations up to 2km in urban areas and 5km in rural areas to protect the confidentiality of respondents [29]; thus, geolinking methods may be less comparable to exact match methods when using DHS or other displaced data. More research of this kind using displaced cluster geolocations and in settings with more dense and complex provider environments would be valuable to help develop these methods.

## CONCLUSIONS

Given the importance for responsive policy and allocation of resources of using effective coverage for monitoring and evaluation for RMNCH, it is essential to have clear, evidence-based guidance on how to measure effective coverage in a valid way [6,30]. To our knowledge, this is the largest study to date to compare ecological and exact match linking methods for measuring quality-adjusted effective coverage, and the only such study to use a global survey programme (MICS) as the source of household data. The results of this study, along with those of Willey et al. and Carter et al. [14,15], suggest that ecological linking may be a feasible and valid approach for estimating quality-adjusted effective coverage when a census of providers is used. The findings also highlight the potential benefit in adjusting for provider type and caseload when implementing ecological linking methods The results suggest that there may be benefit in adding a question on ANC facility type to MICS (facility type is already collected for other services), and questions on caseload to facility surveys. These studies and others have highlighted a number of methodological questions that should be addressed in order to develop guidance on linking methods, including the effect of sampling health providers and including non-facility providers on ecological methods, the stability of provider estimates over time, and the calculation of provider quality scores.
