# Supplementary material for: Linking household survey and health facility data for effective coverage measures: a comparison of ecological and individual linking methods using the Multiple Indicator Cluster Survey in Côte d’Ivoire
Source: J Glob Health. 2018 Sep 19;8(2):020803. doi: 10.7189/jogh.08.020803 (PMC6211616; doi:10.7189/jogh.08.020803)

Online Supplementary Document

Munos et al. Linking household survey and health facility data for effective coverage measures: a comparison of ecological and individual linking methods using the Multiple Indicator Cluster Survey in Côte d'Ivoire

J Glob Health 2018;8:020803

Supplemental Table S1: Components of structural quality indices, by service type

	Antenatal	L&D	Newborn	Postnatal	Sick Child
**Service availability**		• L&D antibiotic administration<br>• L&D function oxytocic for hemorrhage<br>• L&D anticonvulsants<br>• L&D assisted vaginal delivery<br>• L&D manual removal of placenta<br>• L&D removal of retained products<br>• L&D routine practice of Oxytocin for hemorrhage<br>• L&D partographs	• NB breastfeeding<br>• NB cord care<br>• NB thermal protection<br>• NB neonatal resuscitation<br>• NB KMC for LBW babies	• Inpatient<br>• 24-hour staff coverage<br>• FP counseling<br>• Counseling on ITNs<br>• Breastfeeding counseling<br>• Newborn cares counseling<br>• HIV preventive counseling<br>• HIV test counseling<br>• HIV test<br>• ARV to mother<br>• ARV to infant	• General curative care for malaria, ARI, diarrhea<br>• IMCI services<br>• Diagnosis and treatment of malnutrition
**Commodities (drugs, equipment, supplies)**	• ANC dip stick urine protein<br>• ANC dip stick urine glucose	• L&D ambulance<br>• L&D examination light<br>• L&D delivery pack<br>• L&D suction apparatus<br>• L&D manual vacuum extractor	• NB antibiotics for preterm<br>• NB corticosteroids<br>• NB injectable antibiotics	• Emergency transportation<br>• Injectable antibiotics	• Cotrimoxazole or amoxicillin<br>• ACTs<br>• ORS<br>• Zinc

	• ANC urine pregnancy test<br>• ANC iron/folic acid<br>• ANC tetanus toxoid vaccine<br>• ANC IPT drug	• L&D vacuum aspirator or D&C kit<br>• L&D neonatal bag and mask<br>• L&D delivery bed<br>• L&D blank partographs<br>• L&D disposable gloves<br>• L&D infant weighing scale<br>• L&D blood pressure apparatus<br>• L&D soap/hand rub<br>• L&D eye ointment<br>• L&D injectable uterotonic<br>• L&D injectable antibiotics<br>• L&D magnesium sulphate<br>• L&D skin disinfectant<br>• L&D IV solution Ringer Lactate		• Infant weighting scale<br>• Blood pressure apparatus<br>• Thermometer<br>• Stethoscope	• Injectable antibiotics<br>• Injectable antimalarials<br>• Infant/child scale<br>• Thermometer<br>• Stethoscope<br>• Microscopy supplies<br>• RDT<br>• Timer<br>• ORS supplies
**Training, supervision, guidelines**	• ANC trained on screening<br>• ANC trained on counseling<br>• ANC trained on complications<br>• ANC trained on malaria<br>• ANC trained on IPTp<br>• ANC qualified staff<br>• ANC staff supervisions<br>• ANC staff with observed supervision	• L&D guidelines for BEmONC<br>• L&D checklist/job aid<br>• L&D guidelines for essential childbirth observed<br>• L&D qualified staff<br>• L&D trained<br>• L&D guidelines for CEmONC<br>• L&D skilled person 24 hrs<br>• L&D staff supervisions<br>• L&D staff with observed supervision	• NB guidelines for BEmONC	• Trained on counseling for PNC<br>• Trained on management of complications of pregnancy<br>• Trained on PMTCT<br>• Trained on nutrition counseling for newborn of mother with AIDS<br>• Trained on infant and young child feeding for HIV+ mothers<br>• Trained on PMTCT prophylactic treatment	• Trained on IMCI<br>• Trained on iCCM (CHWs)<br>• Trained on malaria diagnosis and management<br>• Trained on ARI diagnosis and management<br>• Trained on diarrhea management<br>• Trained on pediatric HIV diagnosis and management<br>• Trained on nutritional assessment

				• Supervised with observation	• IMCI guidelines observed<br>• Supervised with observation

Supplemental Table S2: Components of process quality indices, by service type

L&D	Immediate newborn	Postnatal	Sick child
• L&D sterile gloves<br>• L&D disinfectant<br>• L&D gauze<br>• L&D clean clothes/towels to dry the baby<br>• L&D cloth to wrap the baby<br>• L&D sterile scissor or new razor blade to cut the cord<br>• L&D cord ligatures<br>• L&D Oxytocine<br>• L&D Ergometrine<br>• L&D Misoprostol<br>• L&D Syntometrine<br>• L&D eye ointment<br>• L&D Chlorhexidine for the newborn cord<br>• L&D monitor labor progress<br>• L&D monitor fetal heartbeat<br>• L&D monitor color of amniotic fluid<br>• L&D monitor degree of molding<br>• L&D monitor dilatation of the cervix<br>• L&D monitor descent of the head	• NB clean baby's mouth before shoulder comes out<br>• NB clean baby's mouth, face and nose<br>• NB ensure the baby is breathing<br>• NB ensure the baby is dry<br>• NB observe for color<br>• NB ensure the baby is kept warm<br>• NB administer prophylaxis for the eyes<br>• NB weigh the baby<br>• NB care for the umbilical cord<br>• NB initiate breastfeed within the first hour<br>• NB evaluate/examine the newborn within the first hour<br>• NB resuscitation open the airways<br>• NB resuscitation clean the mouth/use suction device<br>• NB resuscitation stimulating/drying/wrapping the baby	**History/examination**<br>• Initial assessment of child's health (signs and symptoms since childbirth)<br>• Initial assessment of mother's health (signs and symptoms since delivery)<br>• Other assessments (breastfeeding, child's weight/growth, etc.)<br>• Physical examination of the child<br>• Physical examination of the mother<br>**Preventive treatment**<br>• Iron tablets<br>• Vitamin A capsule<br>• Family planning method<br>**Counseling**<br>• Advice on preventive PNC (keeping baby warm, cord, breastfeeding, FP, postpartum hygiene, ITN)<br>• Advice to seek care if child has any danger sign (fever, cold, difficulty to breastfeed, rapid/difficult breathing, etc.)	• IMCI integrated assessment index<br>○ Checked for 3 danger signs<br>○ Checked for fever, cough/difficult breathing, and diarrhea<br>○ Weighed and weight plotted on growth chart<br>○ Checked for palmar or conjunctival pallor<br>○ Checked health card<br>• Child correctly treated per provider diagnosis

• L&D monitor uterine contraction<br>• L&D monitor maternal blood pressure<br>• L&D monitor maternal temperature<br>• L&D monitor maternal pulse	• NB resuscitation use the ambu bag<br>• NB resuscitation heart massage<br>• NB cord care Chlorhexidine<br>• NB cord care alcohol<br>• NB cord wrapped in dry cloth	• Advice to seek care if mother has any danger sign (sudden and profuse bleeding, vomiting, faintness, fever, etc.)	

Supplemental Table S3: Results of ecological linking, restricting provider dataset to public health facilities

Antenatal care	ANC1				ANC4			
	Structure adjusted				Structure adjusted			
	%	95% CI lower bound	95% CI upper bound	Abs. diff.	%	95% CI lower bound	95% CI upper bound	Abs. diff.
Exact match linking	30.92%	27.09%	34.76%	REF	12.10%	8.86%	15.34%	REF
Ecological linking (Public facilities only)								
Aggregate by district (unweighted)	27.44%	24.80%	30.09%	-3.48	10.49%	8.05%	12.93%	-1.61
Aggregate by district (weighted)	34.87%	31.32%	38.42%	3.94	13.48%	10.21%	16.76%	1.38
Nearest public (straight line)	31.50%	26.90%	36.00%	0.58	13.10%	9.40%	16.80%	1.00
Nearest public (road distance)	31.00%	26.50%	35.40%	0.08	12.80%	9.30%	16.40%	0.70
10 km buffer unweighted	28.50%	24.70%	32.40%	-2.42	11.60%	8.40%	14.80%	-0.50
10 km buffer weighted	30.20%	26.10%	34.30%	-0.72	12.40%	9.00%	15.80%	0.30
Labor & delivery	Structure adjusted				Process adjusted			
	%	95% CI lower bound	95% CI upper bound	Abs. diff	%	95% CI lower bound	95% CI upper bound	Abs. diff
Exact match linking	37.21%	30.48%	43.94%	REF	40.11%	32.88%	47.34%	REF
Ecological linking (Public facilities only)								
Aggregate by district (unweighted)	34.40%	28.56%	40.24%	-2.81	38.57%	31.97%	45.18%	-1.53

Aggregate by district (weighted)	37.91%	31.32%	44.50%	0.70	40.60%	33.57%	47.62%	0.49
Aggregate by district and provider (unweighted)	35.75%	28.99%	42.51%	-1.46	38.38%	31.29%	45.47%	-1.73
Aggregate by district and provider (weighted)	37.54%	30.48%	44.59%	0.33	39.53%	32.22%	46.85%	-0.57
Nearest public (straight line)	37.20%	30.20%	44.20%	-0.01	39.80%	32.20%	47.40%	-0.31
Nearest public (road distance)	37.70%	30.60%	44.80%	0.49	40.60%	32.80%	48.40%	0.49
10 km buffer unweighted	36.80%	30.20%	43.30%	-0.41	40.00%	32.60%	47.40%	-0.11
10 km buffer weighted	38.00%	31.10%	45.00%	0.79	40.60%	33.00%	48.20%	0.49

Immediate newborn	Structure adjusted				Process adjusted			
	%	95% CI lower bound	95% CI upper bound	Abs. diff	%	95% CI lower bound	95% CI upper bound	Abs. diff
Exact match linking	36.14%	28.67%	43.60%	REF	56.46%	46.59%	66.32%	REF
Ecological linking (Public facilities only)								
Aggregate by district (unweighted)	31.69%	26.26%	37.12%	-4.45	55.98%	46.21%	65.74%	-0.48
Aggregate by district (weighted)	38.03%	31.37%	44.69%	1.89	56.03%	46.22%	65.84%	-0.43
Aggregate by district and provider (unweighted)	36.82%	28.92%	44.73%	0.68	53.84%	43.94%	63.73%	-2.62
Aggregate by district and provider (weighted)	38.83%	30.68%	46.99%	2.69	53.96%	44.03%	63.89%	-2.50
Nearest public (straight line)	37.90%	29.50%	46.40%	1.76	55.90%	46.00%	65.70%	-0.56
Nearest public (road distance)	39.10%	30.50%	47.60%	2.96	56.10%	46.10%	66.20%	-0.36
10 km buffer unweighted	36.70%	29.30%	44.20%	0.56	56.20%	46.30%	66.10%	-0.26
10 km buffer weighted	38.40%	30.40%	46.40%	2.26	56.50%	46.50%	66.50%	0.04

PNC for newborns	Structure adjusted				Process adjusted			
	%	95% CI lower bound	95% CI upper bound	Abs. diff	%	95% CI lower bound	95% CI upper bound	Abs. diff
Exact match linking	3.83%	1.07%	6.60%	REF	2.68%	0.78%	4.59%	REF
Ecological linking (Public facilities only)								
Aggregate by district (unweighted)	3.29%	1.06%	5.53%	-0.54	2.56%	0.83%	4.28%	-0.13
Aggregate by district (weighted)	3.97%	1.20%	6.74%	0.13	2.74%	0.89%	4.59%	0.05

Aggregate by district and provider (unweighted)	3.11%	1.10%	5.13%	-0.72	2.20%	0.65%	3.74%	-0.49
Aggregate by district and provider (weighted)	3.50%	1.20%	5.81%	-0.33	2.36%	0.70%	4.01%	-0.33
Nearest public (straight line)	3.60%	1.20%	6.00%	-0.23	2.80%	0.80%	4.70%	0.12
Nearest public (road distance)	3.80%	1.30%	6.20%	-0.03	2.80%	0.80%	4.80%	0.12
10 km buffer unweighted	3.50%	1.10%	5.80%	-0.33	2.70%	0.70%	4.70%	0.02
10 km buffer weighted	3.90%	1.00%	6.90%	0.07	2.80%	0.70%	4.80%	0.12
PNC for mothers	Structure adjusted				Process adjusted			
	%	95% CI lower bound	95% CI upper bound	Abs. diff	%	95% CI lower bound	95% CI upper bound	Abs. diff
Exact match linking	3.21%	0.65%	5.78%	REF	2.14%	0.42%	3.86%	REF
Ecological linking (Public facilities only)								
Aggregate by district (unweighted)	2.56%	0.61%	4.51%	-0.65	1.99%	0.49%	3.48%	-0.15
Aggregate by district (weighted)	3.15%	0.72%	5.58%	-0.06	2.13%	0.53%	3.73%	-0.01
Aggregate by district and provider (unweighted)	2.36%	0.68%	4.04%	-0.86	1.12%	0.14%	2.09%	-1.02
Aggregate by district and provider (weighted)	2.59%	0.75%	4.42%	-0.63	1.20%	0.15%	2.24%	-0.94
Nearest public (straight line)	3.10%	0.90%	5.40%	-0.11	1.90%	0.40%	3.40%	-0.24
Nearest public (road distance)	3.60%	0.80%	6.30%	0.39	1.90%	0.30%	3.50%	-0.24
10 km buffer unweighted	3.20%	0.70%	5.60%	-0.01	2.00%	0.40%	3.60%	-0.14
10 km buffer weighted	3.60%	0.70%	6.40%	0.39	2.00%	0.40%	3.70%	-0.14
Care-seeking for children with fever, diarrhea, or ARI	Structure adjusted				Process adjusted			
	%	95% CI lower bound	95% CI upper bound	Abs. diff.	%	95% CI lower bound	95% CI upper bound	Abs. diff.
Exact match linking	22.90%	18.20%	27.50%	REF	18.30%	14.10%	22.40%	REF
Ecological linking (Public facilities only)								
Aggregate by district (unweighted)	23.38%	19.16%	27.60%	0.48	21.52%	17.68%	25.36%	3.22
Aggregate by district (weighted)	24.76%	20.29%	29.22%	1.86	21.14%	17.37%	24.91%	2.84
Aggregate by district and provider (unweighted)	19.43%	14.72%	24.13%	-3.47	17.40%	13.31%	21.49%	-0.90

Aggregate by district and provider (weighted)	20.18%	15.32%	25.03%	-2.72	17.10%	13.08%	21.12%	-1.20
Nearest public (straight line)	20.10%	15.00%	25.20%	-2.80	16.30%	12.00%	20.60%	-2.00
Nearest public (road distance)	19.40%	14.80%	24.00%	-3.50	16.40%	12.10%	20.70%	-1.90
10 km buffer unweighted	19.50%	14.70%	24.40%	-3.40	16.10%	12.10%	20.10%	-2.20
10 km buffer weighted	19.90%	15.00%	24.80%	-3.00	15.90%	11.80%	19.90%	-2.40

Supplemental Figure S1: ANC structural quality by facility level, managing authority and urban/rural

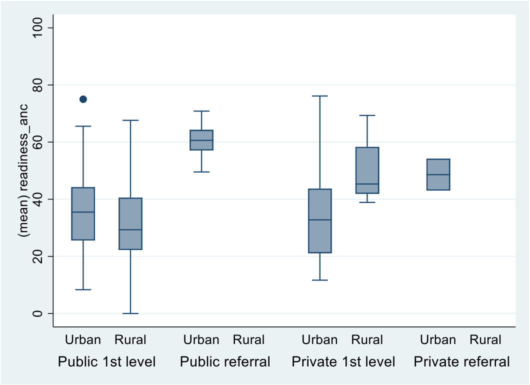

Supplemental Figure S2: ANC structural quality by district and urban/rural

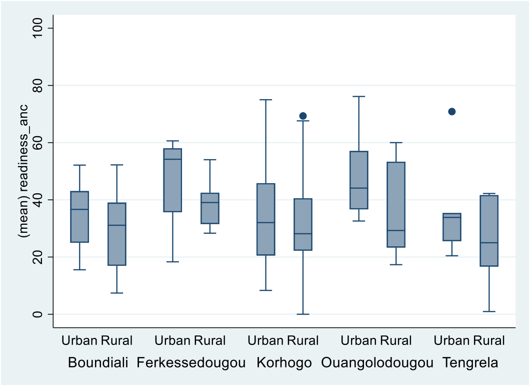

Supplemental Figure S3: Labor & delivery structural and process quality by facility level, managing authority and urban/rural

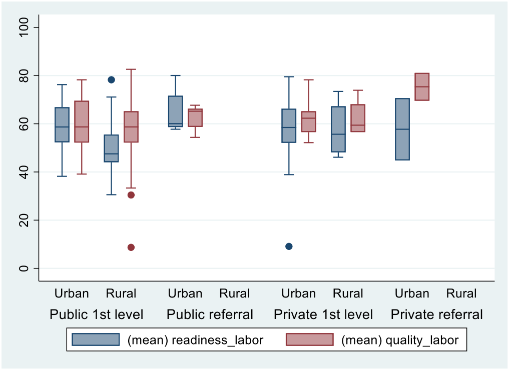

Supplemental Figure S4: Labor & delivery structural and process quality by district and urban/rural

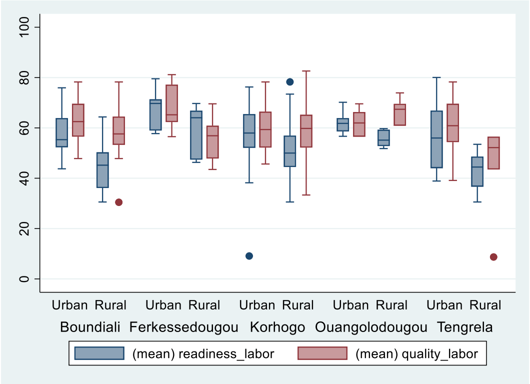

Supplemental Figure S5: Immediate newborn structural and process quality by facility level, managing authority and urban/rural

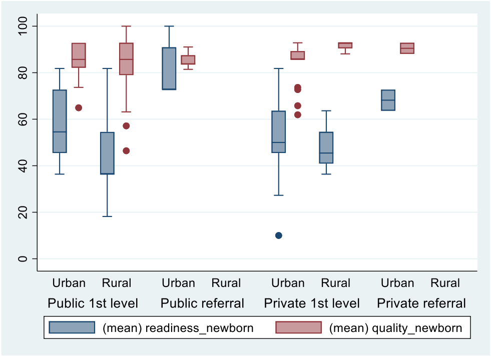

Supplemental Figure S6: Immediate newborn structural and process quality by district and urban/rural

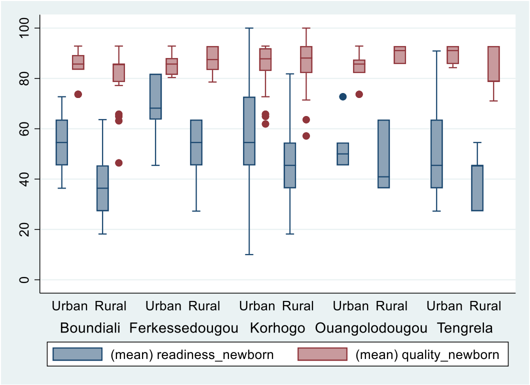

Supplemental Figure S7: PNC structural and process quality by facility level, managing authority and urban/rural

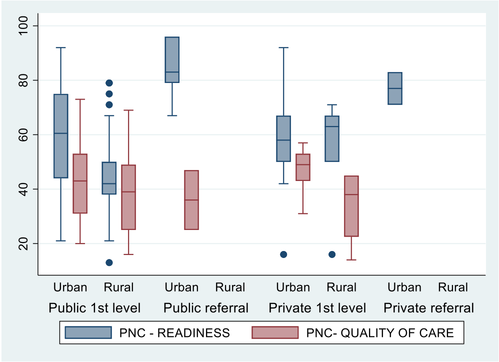

Supplemental Figure S8: PNC structural and process quality by district and urban/rural

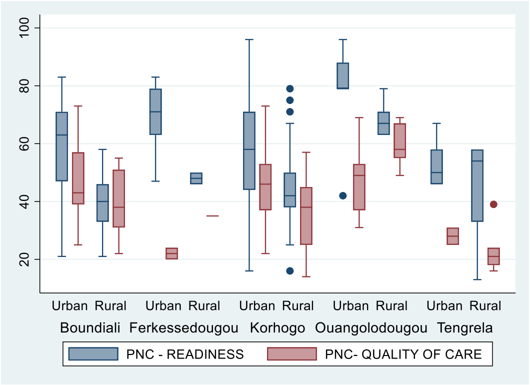

Supplemental Figure S9: Sick child structural and process quality by facility level, managing authority and urban/rural

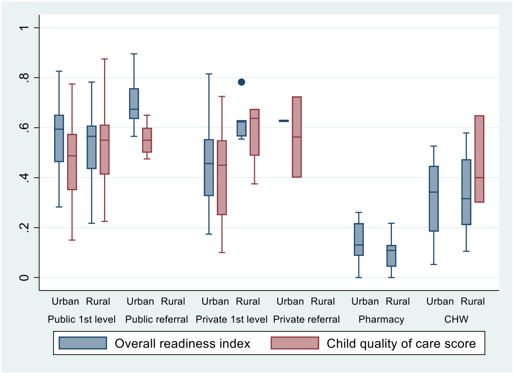

Supplemental Figure S10: Sick child structural and process quality by district and urban/rural

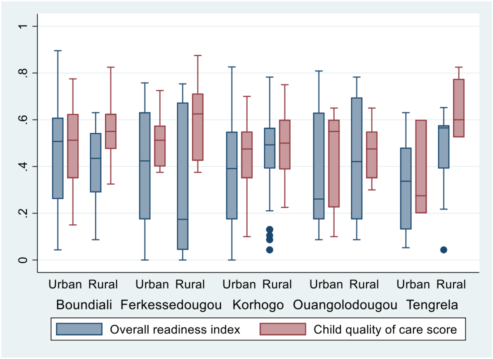

Supplement: Online Supplementary Document [file jogh-08-020803-s001.pdf]
